# A theoretically informed, mixed-methods study of pharmacists’ aspirations and readiness to implement pharmacist prescribing

**DOI:** 10.1007/s11096-021-01296-1

**Published:** 2021-06-14

**Authors:** Derek Stewart, Abdulrouf Pallivalapila, Binny Thomas, Yolande Hanssens, Wessam El Kassem, Zachariah Nazar, Moza Al Hail

**Affiliations:** 1 College of Pharmacy, QU Health, Qatar University, Doha, Qatar; 2 Women’s Wellness and Research Center, Hamad Medical Corporation, Doha, Qatar

**Keywords:** Clinical pharmacy, Framework, Implementation, Mixed methods, Pharmacist, Prescribing

## Abstract

*Background* Studies have highlighted advancing clinical pharmacy practice in Qatar. *Objective* To explore pharmacists’ aspirations and readiness to implement pharmacist prescribing. *Setting* Hamad Medical Corporation (HMC), the main provider of secondary and tertiary care. *Method* A sequential explanatory mixed-methods design. Questionnaire items were derived from the Consolidated Framework of Implementation Research (CFIR), in domains of: awareness/support; readiness; implementation; and facilitators and barriers. Following piloting, all pharmacists (n = 554) were invited to participate. Questionnaire data were analysed using descriptive and inferential statistics with principal component analysis of attitudinal items. Focus groups were recorded, transcribed and analysed using the Framework Approach. *Main outcome measure* Aspirations and readiness to implement pharmacist prescribing. *Results* The response rate was 62.8% (n = 348), with respondents highly supportive of implementation in Qatar (median 4, scale 0–5, extremely supportive). The majority (64.9%, n = 226) considered themselves ready, particularly those more senior (*p* < 0.05) and classifying themselves innovative (*p* < 0.01). Outpatient (72.9%, n = 221 agreeing) and inpatient (71.1%, n = 218 agreeing) HMC settings were those perceived as being most ready. PCA identified 2 components, with ‘personal attributes’ being more positive than ‘prescribing support’. Facilitators were access to records, organizational/management support and the practice environment, with physician resistance and scope of practice as barriers. Focus groups provided explanation, with themes in CFIR domains of innovation characteristics, characteristics of individuals and the inner setting. *Conclusion* HMC pharmacists largely aspire, and consider themselves ready, to be prescribers with inpatient and outpatient settings most ready. CFIR domains and constructs identified as facilitators and barriers should be focus for implementation.

## Impact of findings

- Key CFIR facilitators and barriers identified can be used in planning the implementation of pharmacist prescribing in Qatar and other countries at a similar stage of development
- Implementation should be carefully planned, with attention paid to highlighting the current evidence, the potential to adapt and trial prescribing models and the need for evaluation
- Those pharmacists identified as innovators, with belief in pharmacist prescribing and being at the action stage of change should be considered as priority for training and implementation
- Emphasis needs to be placed on the setting, including access to records, leadership support and communication networks

## Introduction

Prescribing by pharmacists has been implemented in the United Kingdom (UK), Canada, New Zealand and the United States (US) [1–3], with others currently reviewing structures and processes as a precursor to implementation [3]. Program accreditation, training and practice models (e.g. *collaborative* and *supplementary* models, in which there is shared responsibility through collaborative agreements; whereas in the *independent* model an individual clinician is responsible and accountable for the assessment and treatment) vary globally. However, broadly speaking the aims are similar. The primary aim centres on improving patient outcomes while preserving safety, with secondary aims of enhancing access to medicines, making better use of professional skills and saving time for patients and physicians [1–5].

Given the evidence base demonstrating widespread suboptimal doctors’ need for prescribing support [6–10], there is potential for pharmacist prescribers to impact patient care and safety. Systematic reviews and meta-analyses have provided evidence of thoughtful decision-making [11], clinical appropriateness [12–14], effectiveness [13, 14], cost-effectiveness [13], safety [12, 14], quality of life [12], and acceptability [15, 16], compared to physician prescribing. Pharmacists are also integrating these clinical and prescribing services within primary care settings, working alongside physicians and other members of the healthcare team [17–20].

While this evidence can facilitate developments across the globe, almost all studies have been conducted in the western hemisphere. It cannot be assumed that models of care and outcomes can be generalized or translated to countries with markedly different cultures and healthcare systems.

Clinical pharmacy practice in Qatar has advanced in recent years, with pharmacists providing a range of direct patient care and cognitive services, particularly in secondary care [21]. For example, a pharmacist-led anticoagulation clinic of patient assessment, dose adjustment, monitoring and education yielded positive clinical outcomes [22]. Notably, key healthcare stakeholders have recently voiced their support for expanding the clinical scope of pharmacists’ practices [21, 23]. Recent studies have also demonstrated the need to improve medication safety practices generally and prescribing practices specifically [24–27]. Pharmacist prescribing could therefore significantly contribute towards improving health service efficiency and health outcomes, as articulated in Qatar Vision 2030 and the Qatar National Health Strategy 2018–2022 [28, 29].

Prior to developing and implementing novel models of care, there is a need to study readiness for implementation from a number of key perspectives. A systematic review of 65 studies of pharmacist prescribing implementation identified that a minority (n = 29) included pre-implementation investigations [15], and only one study included investigations of both pre- and post-implementation [30]. The review highlighted the lack of attention to implementation theories in design, data collection and reporting, being described in three studies, one of which was pre-implementation [31–33]. The review authors noted the need for implementation studies to incorporate theory thereby enabling more comprehensive investigation of factors likely to impact implementation which could be incorporated into action planning [15]. A recent qualitative study of stakeholders in strategic positions of policy influence in Qatar explored views regarding the potential development and implementation of pharmacist prescribing [23]. Using the Consolidated Framework for Implementation Research (CFIR) [34], a meta-theoretical implementation framework developed from related implementation theories and models; the researchers reported that, while there was support, systematically planning with reference to theoretical domains could remove many potential barriers. More specifically, since the CFIR categorizes implementation determinants in five domains of *intervention characteristics, outer setting, inner setting, characteristics of individuals*, and *planning*; a framework analysis approach facilitated the identification of specific determinants that were considered crucial, namely training, demonstration of competence and engagement of stakeholders [23]. A modified Delphi consensus study with representatives of the same stakeholders used these findings in the development of an implementation framework [35]. A later mixed-methods study of the views of future pharmacists on pharmacist prescribing and its potential implementation yielded support, identified potential barriers of prescribing competence, pharmacist mindset, lack of accessibility to patient records and diversity of education and training [36].

Those who will enact any intervention should be a key focus of any implementation studies. Previous research in Qatar identified that pharmacists working clinically in the national health service (Hamad Medical Corporation, HMC) are likely to be those most ready hence should be a priority for research.

### Aim of the study

The study aimed to explore pharmacists’ aspirations and readiness to implement pharmacist prescribing.

### Ethics approval

Approval was obtained from the Institutional Review Boards of HMC (MRC 01–18-50) and Qatar University (QU-IRB 1431-EA/20).

## Method

### Design

The design was sequential explanatory mixed-methods comprising a cross-sectional survey followed by focus groups providing explanation of survey findings [37].

### Setting

The study was conducted from May-December 2020 across the existing 9 specialist and 3 community hospitals of HMC, the main provider of secondary and tertiary healthcare.

### Cross-sectional survey

#### Questionnaire development

A draft questionnaire was developed based on the literature on development and implementation of pharmacist prescribing [2–5, 15], adapted for the Qatar setting. Items were grouped into the following categories: personal demographics/professional characteristics; support for pharmacist prescribing; readiness to undertake prescribing; implementing prescribing in practice; and facilitators and barriers. Question types were largely closed and Likert scales, with items relating to support, implementation, and facilitators and barriers derived from CFIR [34]. Items were mapped to the CFIR domains of innovation characteristics (of the intervention being implemented), inner setting (i.e. structural, political, and cultural context through which implementation process will proceed) and characteristics of individuals (who will enact the intervention), as these resonated most strongly in previous qualitative research in Qatar [23, 35]. Readiness to become a pharmacist prescriber was categorized according to the Stage of Change model of ‘pre-contemplation’, ‘contemplation’, ‘preparation’ and ‘action’ [38]. For professional characteristics, respondents classified themselves as innovators, early adopters, early majority, late majority and laggards based on receptivity to change [39]. The questionnaire was reviewed for face and content validity by academics, researchers and practicing pharmacists with expertise in pharmacist prescribing. Review for face and content validity was followed by ‘think aloud’ testing with 2 pharmacists to provide an assessment of question clarity from the respondent’s perspective [40]. The questionnaire was then piloted in a sample of 25 pharmacists in HMC, with responses retained as part of the study dataset. Pre-testing findings were incorporated into the final questionnaire which was formatted in SurveyMonkey® and tested for compatibility with platforms (personal computer, tablet, smartphone etc.), browsers and health service email and internet filters. As the common working language at HMC is English, translation into other languages (e.g. Arabic) was not warranted.

#### Recruitment and data collection

An email was sent by the HMC Executive Director of Pharmacy to all pharmacists at the time of the study (n = 554), with no exclusions. Three hundred and sixty responses were required to give a margin of error of 5% with 95% confidence intervals [41]. The email contained a link to the questionnaire and study information outlining the study aim, potential benefits and assuring anonymity. Participation was further encouraged through HMC web alerts and 2 follow-up reminder emails sent at 2-weekly intervals.

#### Analysis

Descriptive analysis was undertaken for: demographics and professional characteristics; support for pharmacist prescribing; readiness to undertake prescribing; implementing prescribing; and facilitators and barriers. Statistically significant differences in scores for supporting pharmacist prescribing in general and specifically in Qatar (0, not supportive at all to 5, extremely supportive) for demographic and professional characteristic variables were tested using Mann–Whitney U test. Each variable was collapsed into two groups (male v female; < 35 years v ≥ 35 years; licensed pharmacists ≤ 20 years v > 20 years; senior position v others; patient contact v no contact; early adopter v others); P-values ≤ 0.05 were considered to be statistically significant. Readiness to undertake prescribing training was summarized into 2 categories: those ‘not ready’ (I have never thought about training as a pharmacist prescriber/I have thought about research training but have taken no action) and the remainder, ‘ready’. Variables significantly associated with readiness were identified using Chi-square.

Five-point Likert scale items relating to implementing prescribing in practice were subjected to principal component analysis (PCA) [42]. Orthogonal (Varimax) rotation was performed to aid in the interpretation of the components, and the results compared to oblique (Promax) rotation. The number of components retained was based on the Kaiser criterion (Eigenvalues > 1), visual inspection of the scree plot and meaningfulness of the results. The analysis included items that were not freestanding, cross-loading or decreasing the scale's internal reliability, and that displayed acceptable communalities, with factor pattern/structure coefficients above 0.4. In performing PCA, the Kaiser–Meyer–Olkin (KMO) measure of sampling adequacy and the Bartlett's Test of Sphericity were used to assess the suitability of the sample for PCA [43]. Where items cross-loaded onto more than one component, the item was captured within the component of highest loading. Following determination of internal consistencies (Cronbach's alpha > 0.60) [44], total scores (median and interquartile range, IQR) were obtained by assigning scores of 1 (strongly disagree) to 5 (strongly agree) to each of the Likert statement responses and each compared to the scale midpoint. Inferential analysis (Mann–Whitney U) was used to explore any relationship between demographics/professional characteristics and component scores.

### Focus groups

To clarify and explain issues identified in the survey phase, pharmacists responding to the questionnaire were invited to participate in an online focus group discussion via Microsoft Teams. Interested participants were requested to add their contact details for follow-up correspondence.

#### Sampling and recruitment

Respondents were purposively sampled in strata of gender, age, and setting, whilst also targeting those with patient-facing roles and high readiness to prescribe.

#### Topic guide development

The topic guide was developed following analysis of questionnaire findings, with the intention of providing further explanation of CFIR domains potentially influencing the implementation of pharmacist prescribing. The topic guide was reviewed for credibility by three members of the research team.

#### Data generation

Focus groups were moderated by two researchers experienced in qualitative research generally and the conduct of focus groups specifically (DS, ZN). Signed, informed consent was obtained from each participant at the outset. Discussions were audio recorded (with permission), transcribed in full and transcripts anonymized, identifying individuals with unique codes. All participants were given the opportunity to review their transcripts, promoting credibility and dependability [45]. Sampling and recruitment continued to the point of data saturation, at which no new themes were generated from the data analysis.

#### Data analysis

Data analysis followed the Framework Approach of: familiarisation; identifying a framework based on CFIR; indexing; charting, and mapping and interpretation [46]. Two experienced qualitative researchers independently coded each focus group, with consensus reached by discussion amongst the research team.

## Results

Three hundred and forty-eight responses were received giving a response rate of 62.8%. Respondents’ personal and practice demographics are given in Table 1. Responses were across the spectrum of roles, largely staff pharmacists (predominantly located in the dispensary) (37.4%, n = 130) and clinical pharmacists (23.6%, n = 82). The majority (83.3%, n = 290) were aged less than 45 years and had been licensed as pharmacists for up to 20 years (81.4%, n = 283). They had obtained their pharmacy qualifications from a range of countries, most frequently Egypt (26.7%, n = 93), India (18.1%, n = 63), Jordan (16.7%, n = 58) and Qatar (11.2%, n = 39). Almost half (48.3%, n = 168) had a graduate qualification and three quarters (75.0%, n = 261) had patient contact. In terms of receptivity to change, most (82.5%, n = 285) classified themselves as innovators or early adopters, and very few (0.9%, n = 3) as laggards.

**Table 1:** Respondent demographics and professional characteristics (n = 348)

Demographic	% (n)
*Current role*^a^	
Director of Pharmacy	1.7 (6)
Assistant Director of Pharmacy	2.0 (7)
Clinical Pharmacy Specialist	5.2 (18)
Clinical Pharmacist	23.6 (82)
Pharmacy Supervisor	9.2 (32)
Senior Pharmacist	18.4 (64)
Staff Pharmacist	37.4 (130)
Medication Safety and Quality Pharmacist	1.1 (4)
Junior Pharmacist	1.1 (4)
Missing	0.3 (1)
*Age (years)*	
≤ 25	3.7 (13)
26–34	35.1 (122)
35–44	44.5 (155)
45–60	15.8 (55)
> 60	0.9 (3)
*Gender*	
Male	52.9 (184)
Female	47.1 (164)
*Nationality*	
Egyptian	25.6 (89)
Indian	16.4 (57)
Jordanian	10.6 (37)
Sudanese	10.3 (36)
Palestinian	9.8 (34)
Qatari	4.6 (16)
Other	22.7 (79)
*Entry to practice degree*	
BSc	60.3 (210)
BPharm	19.5 (68)
MPharm	7.2 (25)
PharmD	12.9 (45)
*Country of entry to practice degree*	
Egypt	26.7 (93)
India	18.1 (63)
Jordan	16.7 (58)
Qatar	11.2 (39)
Sudan	8.3 (29)
Other	19.0 (66)
*Graduate qualifications*^b^	
Any graduate qualification	48.3 (168)
Certificate	10.6 (37)
Diploma	12.4 (34)
MSc	25.9 (90)
MPhil	1.4 (5)
PhD	5.2 (18)
*Years licensed as a pharmacist*	
< 1	2.6 (9)
1–5	12.1 (42)
6–10	22.4 (78)
11–15	23.0 (80)
16–20	21.3 (74)
> 20	18.4 (64)
Missing	0.3 (1)
*Average hours per week with patient contact*	
0	25.0 (87)
1–10	8.9 (31)
11–20	8.3 (29)
21–30	14.1 (49)
31–40	30.2 (105)
> 40	13.5 (47)
*Receptivity to change*	
Willing to take risks in relation to new ways of working	43.7 (152)
Serve as a role model for others in relation to new ways of working	38.8 (135)
Deliberate for some time before adopting new ways of working	11.2 (39)
Cautious in relation to new ways of working; tend to change once most peers have done so	5.5 (19)
Resist new ways of working	0.9 (3)

^a^HMC definitions: Director of Pharmacy, lead for all aspects of the pharmacy department activities; Assistant Director of Pharmacy, Responsible for planning, coordinating and directing all pharmacy activities; Clinical Pharmacy Specialist, possess advanced expertise and experience in clinical pharmacy; Clinical Pharmacist, provide clinical pharmacy services, tend to be less expert and experienced; Pharmacy Supervisor, operationally accountable for managing and delivering aspects of the pharmacy service, largely supply related; Senior Pharmacist, experienced in providing aspects of the pharmacy service; Staff Pharmacist, provides aspects of the pharmacy service; Medication Safety and Quality Pharmacist, particular input to medication safety; Junior Pharmacist, recent graduate undertaking rotational training in all aspects of the pharmacy service

^b^Several respondents had more than one graduate qualification

The majority of respondents (81.3%, n = 283) were aware that, in some countries, pharmacists could legally prescribe medicines which traditionally only physicians could prescribe. They had become aware through a number of different activities, largely attending conferences (45.7%, n = 159), seminars (27.6%, n = 96), workshops (26.4%, n = 92) and university studies (32.2%, n = 112). In terms of specific models of prescribing, respondents were most aware of prescribing by protocol (37.4%, n = 130) followed by collaborative (36.8%, n = 128), supplementary (26.1%, n = 91) and independent (28.4%, n = 99).

On a scale of 0 (not supportive at all) to 5 (extremely supportive), the median rating (interquartile range, IQR) for supporting pharmacist prescribing in general was 4 (3–5) and supporting pharmacist prescribing in Qatar was 4 (3–5). Those with graduate qualifications gave statistically significantly higher ratings for supporting pharmacist prescribing in general (*p* < 0.05, Mann–Whitney U test) but not in Qatar (*p* = 0.145, Mann–Whitney U test). There were no significant differences regarding the other demographic and professional characteristics and support for pharmacist prescribing in general or in Qatar.

Table 2 gives responses in relation to the importance of pharmacist prescribing and readiness of settings to implement pharmacist prescribing within Qatar. The highest levels of agreement were in relation to pharmacist prescribing being important for improving the safe use of medicines (strongly agree/agree 87.8%, n = 267), the economic use of medicines (strongly agree/agree 86.6%, n = 265) and patient care outcomes (strongly agree/agree 84.8%, n = 258). The setting considered most ready was HMC outpatients (strongly agree/agree 72.9%, n = 221), with community pharmacy considered least ready (strongly agree/agree 40.1%, n = 119).

**Table 2:** Responses in relation to importance of pharmacist prescribing and readiness of settings to implement pharmacist prescribing

Pharmacist prescribing in Qatar is important for…	Strongly agree % (n)	Agree % (n)	Neutral % (n)	Disagree % (n)	Strongly disagree % (n)
improving patient care outcomes (n = 304)	40.1 (122)	44.7(136)	11.8 (36)	1.0 (3)	2.3 (7)
improving the safe use of medicines (n = 304)	52.3 (159)	35.5 (108)	8.2 (25)	1.3 (4)	2.6 (8)
improving the economic use of medicines (n = 306)	52.9 (162)	33.7 (103)	9.8 (30)	1.3 (4)	2.3 (7)
patients themselves (n = 294)	37.1 (109)	41.5 (122)	17.3 (51)	2.4 (7)	1.7 (5)
other health professionals (n = 293)	30.4 (89)	43.3 (127)	21.5 (63)	2.7 (8)	2.0 (6)

Setting is ready for pharmacist prescribing	Strongly agree % (n)	Agree % (n)	Neutral % (n)	Disagree % (n)	Strongly disagree % (n)
Hamad Medical Corporation (Inpatient) (n = 304)	34.2 (104)	37.5 (114)	18.4 (56)	7.9 (24)	2.0 (6)
Hamad Medical Corporation (Outpatient) (n = 303)	30.0 (91)	42.9 (130)	17.2 (52)	7.6 (23)	2.3 (7)
primary healthcare clinics (n = 297)	13.1 (39)	32.0 (95)	37.4 (111)	14.8 (44)	2.7 (8)
community pharmacy (n = 297)	13.8 (41)	26.3 (78)	32.0 (95)	19.2 (57)	8.6 (26)

A number of respondents did not complete this section of the questionnaire

In terms of readiness to undertake training, 24 (6.9%) reported already practicing prescribing activities, 202 (58.0%) would be one of the first to undertake prescribing training, 25 (7.2%) would train if other colleagues were also training, 17 (4.9%) would train but only after other colleagues had been practicing as prescribers for a certain period, 22 (6.3%) would think about training but be unlikely to take it any further and 9 (2.6%) would never think about training as a pharmacist prescriber (49 missing responses). There were statistically significant associations between specific demographics and professional characteristics and readiness to undertake prescribing training. Those in more senior positions and those classifying themselves as innovators or early adopters reported being statistically significantly more ready (*p* < 0.05, *p* < 0.01 respectively, Chi square).

**Fig. 1:**
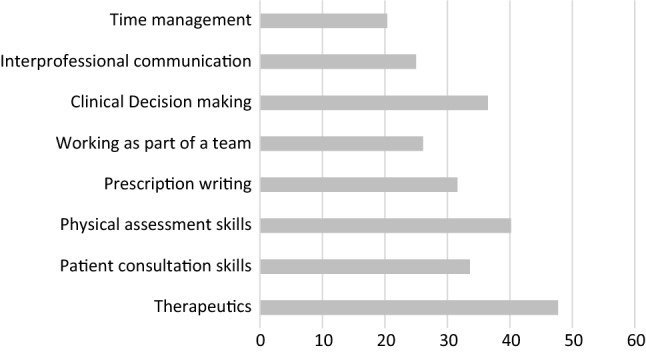
Percentage of respondents citing specific areas of training need

Responses to specific areas of training are given in Fig. 1. The most frequently cited areas of training were therapeutics (47.7%. n = 166), physical assessment skills (40.2%, n = 140) and clinical decision making (36.5%, n = 127, with time management the least frequently cited (20.4%, n = 71).

Levels of agreement with attitudinal statements on pharmacist prescribing are given in Table 3. When these items were subjected to PCA, the correlation matrix contained multiple coefficients above 0.3. The Kaiser–Meyer–Olkin measure of sampling adequacy (0.898) and Bartlett's test of sphericity (significance < 0.001) confirmed the factorability of the items. Two components had Eigenvalues exceeding 1.0, with the two-factor solution explaining 67.9% of the variance. The two components were labelled personal attributes (Cronbach's alpha internal consistency 0.91) and prescribing support (Cronbach's alpha internal consistency 0.79).

**Table 3:** Levels of agreement with attitudinal statements on pharmacist prescribing

	Strongly agree % (n)	Agree % (n)	Neutral % (n)	Disagree % (n)	Strongly disagree % (n)
*Component 1—personal attributes*					
It is my professional duty to become a pharmacist prescriber (n = 294)	31.3 (92)	43.5 (128)	20.7 (61)	3.4 (10)	1.0 (3)
Practicing as a pharmacist prescriber would improve the care of my patients (n = 295)	44.7 (132)	46.8 (138)	7.1 (21)	0.7 (2)	0.7 (2)
A pharmacist prescriber role would enhance my professional image (n = 295)	56.3 (166)	34.9 (103)	6.4 (19)	1.4 (4)	1.0 (3)
I already have access to all the patient information I need to practice as a pharmacist prescriber (n = 294)	37.1 (109)	37.4 (110)	19.0 (56)	4.8 (14)	1.7 (5)
Pharmacist prescribing would work well in my setting (n = 293)	33.1 (97)	44.4 (130)	17.7 (52)	3.8 (11)	1.0 (3)
I would be happy to become a pharmacist prescriber (n = 293)	53.9 (158)	36.2 (106)	7.8 (23)	1.0 (3)	1.0 (3)
I am confident in my ability to become a pharmacist prescriber (n = 293)	50.2 (147)	40.3 (118)	6.8 (20)	2.0 (6)	0.7 (2)
Component statistics, sum of allocating 1 (strongly disagree) to 5 (strongly agree) Cronbach's alpha 0.91 Range possible 7–35, with 35 representing best positive score Mid-point 21 Median 30 IQR 27–33					
*Component 2—prescribing support*					
I have sufficient administrative support to implement pharmacist prescribing (n = 294)	22.8 (67)	31.6 (93)	34.0 (100)	8.5 (25)	3.1 (9)
I have sufficient IT support to implement pharmacist prescribing (n = 294)	23.1 (68)	37.4 (110)	32.7 (96)	5.8 (17)	1.0 (3)
I have sufficient pharmacist and technical support to implement pharmacist prescribing (n = 293)	22.9 (67)	39.2 (115)	29.7 (87)	5.1 (15)	3.1 (9)
Component statistics, sum of allocating 1 (strongly disagree) to 5 (strongly agree) Cronbach's alpha 0.79 Range possible 3–15, with 15 representing best positive score Mid-point 9 Median 11 IQR 9–13					

A number of respondents did not complete this section of the questionnaire

For component 1, personal attributes, respondents generally held very positive views, with a median overall score of 30 (IQR 27–33), range possible 7–35 (midpoint 21), with 35 representing the highest possible positive score. The statement with the highest level of agreement was, ‘Practicing as a pharmacist prescriber would improve the care of my patients’ (agree/strongly agree n = 270, 91.5%) and that with the lowest level of agreement was, ‘I already have access to all the patient information I need to practice as a pharmacist prescriber’ (agree/strongly agree n = 219, 74.5%). Component 1 scores were statistically significantly higher for males compared to females (median 31v29, Mann–Whitney U, *p* < 0.05), for those with patient contact (median 31v28.5, Mann–Whitney U, *p* = 0.001) and those classifying themselves as innovators or early adopters (median 31v28, Mann–Whitney U, *p* < 0.05).

For component 2, prescribing support, respondents generally held much more neutral views, with a median overall score of 11 (IQR 9–13), range possible 3–15 (midpoint 9), with 15 representing the highest possible positive score. The statement with the highest level of agreement was, ‘I have sufficient pharmacist and technical support to implement pharmacist prescribing’ (agree/strongly agree n = 182, 62.1%) and that with the lowest level of agreement was, ‘I have sufficient administrative support to implement pharmacist prescribing’ (agree/strongly agree n = 160, 54.4%). Component 2 scores were statistically significantly higher for males compared to females (median 12v10, Mann–Whitney U, *p* < 0.001) but there were no statistically significant differences for any other demographic or professional characteristics.

**Fig. 2:**
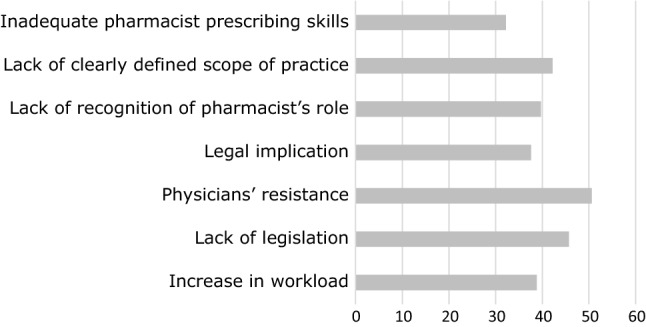
Percentage of respondents selecting specific barriers to implementing pharmacist prescribing

**Fig. 3:**
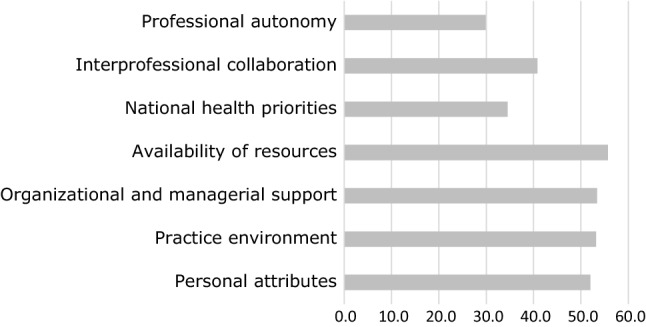
Percentage of respondents citing specific facilitators to implementing pharmacist prescribing

Responses to specific barriers and facilitators to implementing prescribing are given in Figs. 2 and 3 respectively. The most frequently cited barriers were physicians’ resistance (50.6%, n = 176), lack of legislation (45.7%, n = 159) and lack of clearly defined scope of practice (42.2%, n = 147), with inadequate prescribing skills the least frequently cited (32.2%, n = 112). The most frequently cited facilitators were availability of resources such as workspace and access to the medical record (55.7%, n = 194), organizational and managerial support (53.4%, n = 186) and the practice environment (53.2%, n = 185), with professional autonomy the least frequently cited (29.9%, n = 104).

One hundred and sixty-two respondents (46.6%) expressed interest in participating in the qualitative study. Of these, 100 provided full contact details, had patient contact and classified themselves as innovators or early adopters hence were eligible for participation. Three focus groups were conducted (50–75 min in duration), each consisting of pharmacists of different grades, roles and years of experience. It was deemed that data saturation of the themes had been achieved after the third focus group.

The analysis of the focus group discussions focused on identifying explanations and clarifications of issues identified in the survey phase. Table 4 provides the key themes that emerged which were mapped to the CFIR implementation determinants. Illustrative quotes are provided for each. The following commentary has been extracted from the focus group analysis to clarify and explain issues identified in the survey phase.

**Table 4:** Focus group themes and illustrative quotes mapped to CFIR domains and constructs

CFIR Domain	CFIR constructs	Themes	Illustrative quotes
Innovation characteristics	Innovation source	Good awareness of pharmacist prescribing and knowledge of the various models	“I’m aware that in South Africa they had an example (of pharmacist independent prescribing)….I know that in Alberta (Canada) there is a form of collaborative prescribing” P1FG3
	Evidence strength and quality	Positive perceptions of existing evidence supporting pharmacist prescribing in other countries	“It’s working in other countries around the world and helps the health system and gives patients better access” P1FG2
	Relative advantage	Initial resistance within the multi-disciplinary team would diminish with exposure to the benefits	“there will be some resistance…not full cooperation.. but there will be a transition when they (physicians) see that it will save them more time and will help them with their prescribing” P3FG3
	Adaptability	Pharmacists have the necessary knowledge and skills to prescribe for minor diseases	“all pharmacists are already competent to prescribe for minor disease, like fever or pain” P4FG3
		Review of workflow and job roles necessary to avoid over-burdening pharmacists	“we could do this but it would be too much with our jobs we have…. They (management) would need to relieve us of some duties” P1FG3
	Trialability	Workflow in outpatient settings is conducive for initial implementation before wider role-out	“start with the outpatients, you can start with prescribing the refills and OTC medications and then look at inpatient settings after” P2FG2
Characteristics of individuals	Knowledge and beliefs about the intervention	Facilitate role expansion and utilize increased existing skills	“we don’t want just to approve refills, we have the skills and knowledge to do more” P4FG2
	Self-efficacy	Previous successes with pharmacist-lead initiatives	“we can identify things that even physicians in a very specialized area, cannot recognize it” P2FG2
	Individual stage of change	Motivated to become prescribers	“we want to be active members, doing active interventions for the therapy and planning for the therapy” P2FG1
Inner setting	Structural characteristics	Existing integration of clinical pharmacists in the multidisciplinary team will facilitate implementation	“the role the clinical pharmacist has with medication management is known and accepted” P3FG1
	Networks and communications	Education and awareness of the pharmacist role is lacking	“in some settings the team doesn’t have awareness of the capabilities of the pharmacist and how they can contribute to the therapeutic plan” P5FG3
	Culture	Physicians are protective of their role and will object to pharmacist role in diagnosis	“they (physicians) will be very sensitive if they see any pharmacists trying to diagnose” P3FG1
		Members of the multidisciplinary team are likely to be very supportive	“there is already support from nurses and lead nurses… diabetic educators and respiratory technicians see us as the medicines experts” P4FG2
	Implementation climate	Setting where clinical pharmacy practice is established will be more ready	“a physician working in an inpatient setting… they will immediately agree with our (pharmacists) recommendations, because they have work with us for many years” P5FG2
		The national health strategy is supportive of expanding the pharmacists’ role	“The national health strategy wants pharmacist-lead clinics and pharmacist prescribing… this will help us” P2FG3

### Innovation characteristics

Consistent with the survey data indicating support for pharmacist prescribing, focus group participants were well-informed and positively perceived the various pharmacist prescribing models. Participants voiced a belief that the benefits reported in the literature could be achieved in HMC settings following a review of existing pharmacist responsibilities. They anticipated a transition period, in which physician resistance may be apparent, but would lessen with increasing exposure to the advantages for patient care and reducing workload. Further, it was felt that although a collaborative pharmacist prescribing model could eventually be common practice at HMC; an initial role-out in settings in which the role of the clinical pharmacist is already well-established may be an effective approach before wider implementation.

### Characteristics of individuals

The survey analysis indicated that pharmacists classified themselves as innovators equipped with good skills; this finding was confirmed in the focus groups. Participants described medication management initiatives they had either lead or contributed to, which had resulted in improved patient care. Involvement in these successful initiatives had a positive impact on self-efficacy and motivation to further their role.

### Inner setting

Although physician resistance was cited most frequently as a potential barrier in the survey responses, focus group analysis revealed a general agreement amongst participants that this resistance was likely to be limited to pharmacists taking a role in diagnosis. Otherwise, it was considered that in settings where there was good knowledge and awareness of the pharmacists’ role, there would be broad acceptance of pharmacists having an enhanced role in medication management within the multidisciplinary team.

## Discussion

Questionnaire respondents were highly supportive of pharmacist prescribing and its implementation in Qatar. The majority considered themselves ready to undertake prescribing, particularly those in senior positions and classifying themselves innovators or early adopters. Outpatient and inpatient HMC settings were those deemed most ready. PCA identified 2 components, with that relating to personal attributes around prescribing having more positive responses compared to support to implement prescribing (e.g. administrative support). Facilitators to implementation were access to medical records, organizational and management support and the practice environment, with barriers relating to physician resistance, and current legislation and scope of practice. Focus groups provided explanation of these findings, with themes in CFIR domains of innovation characteristics (source, evidence strength and quality, advantage, adaptability, trialability), characteristics of individuals (beliefs, self-efficacy, stages of change) and the inner setting (structural characteristics, networks and communications, culture, implementation climate).

There are a number of strengths to this study. As noted earlier, there is a lack of theory informed pre-implementation studies; the use of CFIR is likely to have provided comprehensive coverage of related issues. The sequential, explanatory mixed-methods approach allowed both quantification and explanation of pharmacists’ perspectives [37]. The response rate of almost two thirds of the study population enhances generalizability to all pharmacists within HMC and the attention to aspects of research trustworthiness in the qualitative phase increases study rigour [45]. There are, however, weaknesses specifically around the potential lack of generalizability and transferability of the findings to the Middle East and beyond.

The findings of this study add to the accumulating positive evidence supporting the development and implementation of pharmacist prescribing in secondary care in Qatar [23, 35, 36], and the wider global setting. The use of CFIR in this study has allowed elucidation of key issues to be considered in developments to increase the likelihood of successful implementation, which can then be studied post-implementation.

Questionnaire items on support, implementation, and barriers and facilitators were derived from CFIR domains of innovation characteristics, inner setting and characteristics of individuals, the findings of which were explored in focus groups. In terms of innovation characteristics, while there was high awareness of global developments in pharmacist prescribing, there was less awareness of specific prescribing models. Potential advantages of pharmacist prescribing were described in relation to safety, effectiveness and cost-effectiveness and consequences for health professionals. Further explanation in focus groups highlighted the positive evidence (published and anecdotal) on pharmacist prescribing. There was acknowledgement of the need to trial and adapt prescribing models to the local context, with consideration of specific additional workload and responsibilities, prior to implementation on a wider scale. It was also noted that any initial resistance from physicians would diminish as local evidence emerged. While a number of the findings are similar to other studies, most have reported these post-implementation, having experienced some negative impact on service planning and delivery [15]. Identifying these pre-implementation in Qatar provides an opportunity to account for these at this stage potentially leading to more seamless implementation.

Survey results aligned to the CFIR domain of characteristics of individuals were largely positive with respondents supportive of pharmacist prescribing and potential implementation in Qatar. The results of PCA component 1 (personal attributes) were also particularly positive in relation to perceptions of self-efficacy and benefits to self, patients and others. Indeed, analysis of readiness identified the majority of respondents at ‘action’, ‘preparation’ stages of change. Statistically significant relationships of those with graduate qualifications being more supportive, those senior and innovative being more ready, and those with patient contact and innovative having more positive PCA scores are unsurprising. These individuals are likely to be amongst the first cohort applicants for training. Qualitative findings provided explanation in terms of beliefs of the intervention and role expansion, self-efficacy and previous success, and stages of change and motivation. Specific training needs expressed around therapeutics, physical assessment and decision making are similar to those previously reported [1, 2, 36, 47], and are included in the curricula of many pharmacist prescribing programs.

For the CFIR domain of inner setting, survey respondents perceived the secondary care setting to be more ready than primary care or community pharmacy. This has also been the experience in other countries with pharmacist prescribers in community pharmacy less likely to be using their prescribing qualification [1, 2]. Further, it has been reported that community pharmacies in Qatar mainly focus on traditional pharmacist’s product-oriented role of drugs dispensing [21]. It is, however, notable that PCA component 2 (prescribing support) scores were rather neutral around administrative, pharmacist and technician, and IT support, all aligning with the CFIR construct of readiness for implementation (resources). Key inner setting facilitators highlighted were access to records (resources), organizational and managerial support (leadership engagement) and the practice environment (culture, implementation climate). Barriers were around CFIR inner setting constructs of compatibility and relative priority (physicians’ resistance and legislation). Explanation in the qualitative research extended CFIR constructs in areas of existing integration in, and support from, the multidisciplinary team (structural characteristics), and established practice and developments in line with national strategy (implementation climate). Previous studies have also highlighted existing working relationships, professional respect and trust being key in the rapid implementation of pharmacist prescribing post-training [15]. Other studies have also shown that physician (and other) resistance diminishes on exposure to pharmacist prescribing practice [15].

The findings of this study can be incorporated into development and implementation plans. Use of CFIR has provided a comprehensive understanding of issues to be considered which in turn is likely to lead to more effective and efficient implementation. The CFIR domain of process highlights key constructs of planning, engaging others (opinion leaders, implementation leaders, change champions), executing and reflecting.

Further research will focus on systematically planning and researching implementation using quantitative, qualitative and mixed-methods approaches.

## Conclusion

HMC pharmacists largely aspire to, and consider themselves ready, to be pharmacist prescribers with inpatient and outpatient settings most ready for implementation. There is a need to consider CFIR domains and constructs identified as facilitators and barriers as implementation is planned.
